# Cellular senescence and inflammageing: from mechanisms to senotherapeutic interventions

**DOI:** 10.1007/s10522-026-10477-2

**Published:** 2026-07-23

**Authors:** Piotr Paweł Chmielewski

**Affiliations:** Division of Anatomy, Department of Human Morphology and Embryology, Faculty of Medicine, Wroclaw Medical University, 6a Chałubińskiego Street, 50-368, Wrocław, Poland

**Keywords:** Cellular senescence, Senescence-associated secretory phenotype (SASP), Senotherapeutics, Senolytics, Inflammageing, Biomarkers of senescence, Geroscience

## Abstract

Cellular senescence is a context-dependent cellular state characterised by persistent cell-cycle arrest, epigenetic remodelling, metabolic reprogramming and acquisition of a senescence-associated secretory phenotype. Transient senescence contributes to embryogenesis, tissue repair and tumour suppression, whereas persistent senescent cell populations accumulate with advancing age across multiple tissues, in part owing to declining immune-mediated clearance and intrinsic resistance to apoptosis, thereby promoting chronic systemic inflammation, tissue fibrosis, stem-cell dysfunction and propagation of secondary senescence. Experimental genetic and pharmacological evidence supports a contributory and in several contexts causal role for senescent cells in cardiovascular, metabolic, musculoskeletal, fibrotic and neurodegenerative disorders. These findings have accelerated the development of senotherapeutic strategies, including senolytics, senomorphics and immune-mediated clearance approaches, with early clinical studies showing preliminary evidence of functional benefit in idiopathic pulmonary fibrosis and diabetic kidney disease. However, clinical translation remains constrained by senescence heterogeneity, limited biomarker specificity and unresolved long-term safety concerns. Improved molecular, spatial and functional resolution of senescent states will be essential for developing biomarker-guided and tissue-specific interventions that preserve the beneficial functions of transient senescence while limiting its chronic deleterious effects.

## Introduction

**Fig. 1:**
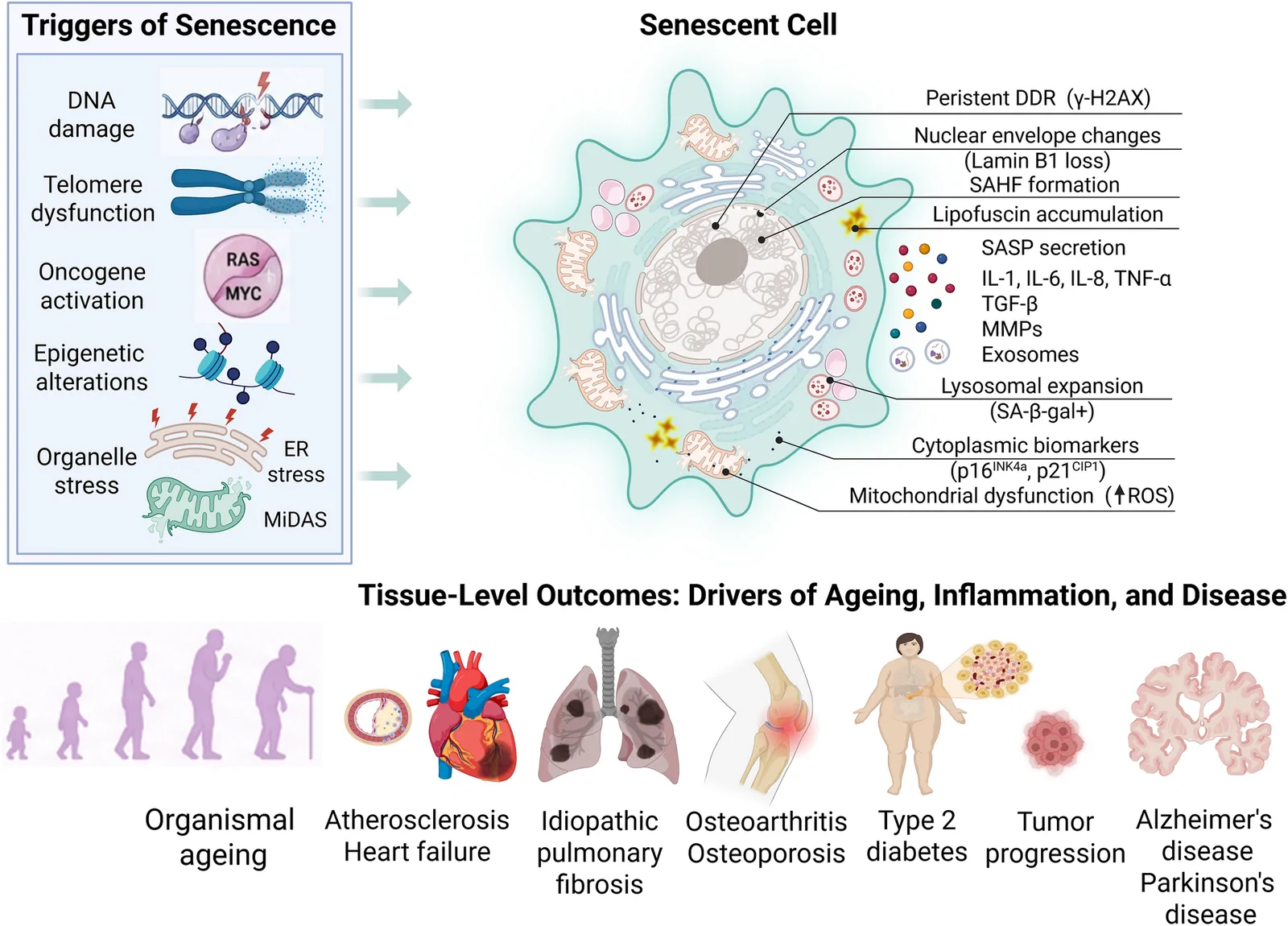
Hallmarks and pathological consequences of cellular senescence during ageing. Multiple stressors, including DNA damage, telomere dysfunction, oncogenic signalling, chromatin remodelling and organelle stress, can induce cellular senescence through activation of cell-cycle inhibitory pathways and establishment of a characteristic senescent phenotype. Senescent cells exhibit persistent DDR signalling, chromatin and metabolic alterations, mitochondrial dysfunction and increased lysosomal activity, together with acquisition of the SASP. Over time, accumulation of senescent cells and persistent SASP signalling contribute to inflammageing, impair tissue function and promote biological ageing as well as multiple age-related pathologies, including cardiovascular, metabolic, musculoskeletal, neoplastic and neurodegenerative diseases. *DDR* DNA damage response, *ER* endoplasmic reticulum, *MYC* MYC proto-oncogene, *MMPs* matrix metalloproteinases, *RAS* RAS proto-oncogene family, *ROS* reactive oxygen species, *SA-β-gal* senescence-associated β-galactosidase, *SAHF* senescence-associated heterochromatin foci, *SASP* senescence-associated secretory phenotype

Originally described by Leonard Hayflick and Paul Moorhead as the finite replicative capacity of human fibroblasts in cell culture (Hayflick and Moorhead 1961; Hayflick 1965), cellular senescence has become a central concept in ageing biology (Rattan 2016; He and Sharpless 2017; Gorgoulis et al. 2019; Borghesan et al. 2020; López-Otín et al. 2023). Cellular senescence is a biologically heterogeneous and context-dependent cellular state with both adaptive and pathological consequences (Rodier and Campisi 2011; Demaria et al. 2014; Muñoz-Espín and Serrano 2014; Regulski 2017), characterised by durable cell-cycle arrest, epigenetic remodelling, metabolic reprogramming and, frequently, acquisition of a senescence-associated secretory phenotype (SASP) (Aird et al. 2016; Hernandez-Segura et al. 2018; McHugh and Gil 2018; Basisty et al. 2020; Wiley and Campisi 2021; Ajoolabady et al. 2025). Senescence-associated growth arrest is established and maintained predominantly through the canonical tumour-suppressor pathways p53–p21^CIP1/WAF1^ and p16^INK4a^–pRB in response to diverse cellular stressors, including DNA damage, telomere dysfunction, oncogene activation, epigenetic alterations, mitochondrial dysfunction and endoplasmic reticulum (ER) stress (Fig. 1).

A useful conceptual distinction can be made between physiological cellular senescence and damage-associated cellular ageing, the latter reflecting the progressive accumulation of molecular and cellular damage accompanied by declining adaptive and repair capacity (Ogrodnik 2021). In physiological contexts, including embryonic development, wound healing and tissue remodelling, cellular senescence is induced in a spatially and temporally regulated manner, contributes to tissue patterning and repair through paracrine signalling and is followed by efficient immune-mediated clearance (Muñoz-Espín et al. 2013). By contrast, during ageing, senescent cells progressively accumulate across tissues owing to persistent cellular stress and damage, declining immune surveillance and intrinsic resistance to apoptosis (Childs et al. 2015; Baker et al. 2016). This distinction is important because it separates context-dependent and beneficial functions of senescence from its maladaptive late-life consequences, which are consistent with antagonistic pleiotropy and evolved constraints on somatic maintenance (Kowald et al. 2020).

The deleterious effects of persistent senescent cells are mediated predominantly through the SASP, which is a dynamic and context-dependent repertoire of pro-inflammatory cytokines, chemokines, growth factors (GFs), proteases and extracellular matrix (ECM) remodelling molecules that disrupt tissue homeostasis, propagate secondary senescence via paracrine signalling and modulate local immune function (Coppé et al. 2010; Nelson et al. 2012; Acosta et al. 2013; da Silva et al. 2019; Wang et al. 2024a, b). In particular, persistent SASP signalling constitutes a major contributor to inflammageing, defined as a chronic, low-grade, sterile inflammatory state arising during biological ageing (Franceschi and Campisi 2014). In contrast to chronic inflammation more broadly, which may arise from diverse sources including metabolic dysfunction, gut dysbiosis, barrier failure and accumulation of damage-associated molecular patterns (DAMPs), inflammageing denotes a multifactorial, system-level inflammatory state in which senescent cell burden represents a central but not exclusive contributor (Krabbe et al. 2004; Vasto et al. 2007). Thus, senescent cells should be regarded as a principal source rather than the sole cause of inflammageing.

Empirical evidence supports a causal role for cellular senescence in organismal ageing and multiple age-related diseases (Baker et al. 2016; Xu et al. 2018; Hu et al. 2022; Ali et al. 2024; Kumar et al. 2024; Rim et al. 2024; McHugh et al. 2025; Melo Dos Santos et al. 2024; Xu et al. 2025). Accordingly, the selective clearance of senescent cells has emerged as a promising therapeutic strategy. By reducing senescent cell burden, senolytics and senomorphics may attenuate chronic systemic inflammation and modulate other hallmarks of ageing, thereby mitigating the risk of multiple age-related pathologies (López-Otín et al. 2023). This rationale is strengthened by shared molecular features of senescent cells, including increased p16^INK4a^, p21^CIP1/WAF1^ and senescence-associated β-galactosidase (SA-β-gal) activity, which may enable cross-tissue targeting. Experimental clearance of senescent cells in vivo delays multiple age-related pathologies and extends lifespan (Baker et al. 2011, 2016). Collectively, these findings establish cellular senescence as a mechanistically actionable therapeutic target and have accelerated the development of senotherapeutic strategies (Gorgoulis et al. 2019; Kirkland and Tchkonia 2020; Saccon et al. 2021; Morsli et al. 2022; Dhokia et al. 2024).

In this perspective, the biological basis of cellular senescence is outlined, the challenges of its in vivo identification and quantification are discussed, and emerging therapeutic strategies to selectively eliminate senescent cells or modulate their pro-inflammatory secretome are critically evaluated, with the aim of translating mechanistic insight into interventions that extend healthspan and lifespan.

## Identifying the senescent phenotype: markers and methodologies

Currently, no single biomarker reliably and specifically defines cellular senescence across tissues, cell types and disease contexts (Gorgoulis et al. 2019; Di Micco et al. 2021; Sikora et al. 2021). Accordingly, robust identification requires a tissue- and context-aware multiparametric approach integrating molecular, structural and functional features, including persistent DNA damage response (DDR) signalling, altered cell morphology, lysosomal expansion, chromatin remodelling and secretion of SASP components.

Molecular markers involved in cell-cycle regulation constitute a core component of senescence detection. The cyclin-dependent kinase inhibitors p16^INK4a^ (encoded by *CDKN2A*) and p21^CIP1/WAF1^ (encoded by *CDKN1A*) are among the most widely used senescence-associated markers (Mirzayans et al. 2012; Gorgoulis et al. 2019; Di Micco et al. 2021). The *CDKN2A* locus also encodes the tumour suppressor p14^ARF^ (p19^Arf^ in mice), which participates in p53-associated signalling (Baker et al. 2008). Although p16^INK4a^ expression increases with age and is elevated in senescent cells induced by diverse stressors (Mirzayans et al. 2012), it is not restricted to senescence and can also occur during terminal differentiation and inflammatory states. Similarly, p21^CIP1/WAF1^ mediates p53-dependent cell-cycle arrest and is rapidly induced following acute DNA damage, but its expression may decline in established senescent cells, limiting its utility for detecting chronic senescence. Loss of lamin B1, a structural component of the nuclear lamina, occurs consistently in many senescence contexts, contributes to chromatin reorganisation and facilitates SASP activation, making it an informative although not fully specific marker (Freund et al. 2012; Matias et al. 2022). Reduced Ki-67 expression can additionally support identification of proliferative arrest.

Persistent DDR signalling is a characteristic feature of many senescent cells and can be inferred from markers such as γH2AX and 53BP1 foci (Jackson and Bartek 2009; Ciccia and Elledge 2010; Nakamura et al. 2010; Hernandez-Segura et al. 2018). γH2AX marks DNA double-strand breaks and may form persistent nuclear structures termed DNA segments with chromatin alterations reinforcing senescence (DNA-SCARS). Their colocalisation with DDR proteins including 53BP1, ATM and MDC1 can help distinguish persistent senescence-associated signalling from transient DNA repair responses. Telomere dysfunction-induced foci (TIFs), identified by colocalisation of γH2AX or 53BP1 with telomeric DNA, provide evidence of telomere-associated senescence.

Morphological and structural alterations provide additional supportive markers. Senescent cells commonly display cellular hypertrophy, flattened and irregular morphology, nuclear enlargement, increased cytoplasmic granularity and expansion of lysosomal compartments (Mitsui and Schneider 1976; Hernandez-Segura et al. 2018; Gorgoulis et al. 2019; Di Micco et al. 2021; Sikora et al. 2021). Nuclear architecture undergoes extensive remodelling, including alterations in heterochromatin organisation, context-dependent formation of senescence-associated heterochromatin foci (SAHFs) and chromatin reconfiguration that may facilitate transcription of SASP-associated genes. Lipofuscin, an autofluorescent and poorly degradable lipid-protein aggregate generated through incomplete lysosomal degradation, accumulates with ageing, particularly in long-lived post-mitotic cells such as neurons and cardiomyocytes (Gray and Woulfe 2005; Jung et al. 2007). SA-β-gal activity remains widely used because of its simplicity and applicability to cultured cells and tissue specimens. However, SA-β-gal activity lacks specificity as a senescence marker because it primarily reflects increased lysosomal content and activity rather than senescence itself (Yang and Hu 2005; Adewoye et al. 2020). Consequently, it may also be detected in quiescent, confluent, differentiated or damaged cells and is not uniformly present across all senescent cell populations.

Functional and secretory phenotypes provide dynamic readouts of cellular senescence. Resistance to apoptosis, mediated by increased expression of BCL-2 family proteins and activation of pro-survival pathways such as PI3K/AKT and NF-κB, represents a common and therapeutically exploitable feature of senescent cells. SASP secretion, quantifiable through ELISA, multiplex cytokine arrays and transcriptomic analyses, provides a functionally relevant marker. The SASP is a dynamic and heterogeneous feature of senescent cells that is regulated by interconnected signalling networks, including NF-κB, C/EBPβ, mTOR and p38 MAPK pathways, with additional contributions from cGAS-STING signalling in many contexts, particularly during DNA damage-associated senescence (Gorgoulis et al. 2019; Di Micco et al. 2021; Sikora et al. 2021). Its components include cytokines, chemokines, GFs, matrix-remodelling enzymes and extracellular vesicles (EVs) that reshape tissue microenvironments (TME) and recruit immune cells.

Importantly, SASP composition varies substantially according to cell type, senescence-inducing stimulus and tissue context (Basisty et al. 2020). For example, senescent fibroblasts frequently secrete IL-6 and IL-8, whereas senescent endothelial cells show enrichment of factors including von Willebrand factor (vWF) and plasminogen activator inhibitor-1 (PAI-1). Context-specific SASP profiling is therefore essential for interpretation and therapeutic targeting. While transient SASP activity can promote immune-mediated clearance and tissue repair, persistent SASP signalling becomes self-amplifying and contributes to inflammageing and age-related pathology.

Emerging technologies are expanding senescence detection capabilities. Single-cell RNA sequencing enables unbiased transcriptomic profiling of senescent subpopulations within heterogeneous tissues, revealing previously unrecognised heterogeneity and candidate biomarkers. Spatial transcriptomics and multiplexed immunofluorescence permit localisation of senescent cells within intact tissue architecture. Senescence-targeted molecular imaging probes, including fluorescently labelled senolytic compounds and activatable reporters responsive to SA-β-gal or γ-glutamyltransferase activity, enable non-invasive visualisation of senescent cells in living systems. Liquid biopsy approaches quantifying circulating SASP factors, senescent-cell-derived EVs or cell-free DNA carrying senescence-associated signatures are also being explored as minimally invasive biomarkers.

To date, no single or composite biomarker has demonstrated the analytical validity, clinical validity and clinical utility required for routine clinical assessment of senescent cell burden. The lack of a universally accepted reference standard for in *vivo* identification of senescent cells fundamentally constrains biomarker validation and precludes robust inter-study comparison. Existing candidates therefore function as surrogate indices of senescence-associated biology rather than direct measures of senescent cell burden or tissue distribution (Muthamil et al. 2024). Circulating SASP mediators, including pro-inflammatory cytokines and chemokines (IL-6, IL-8, TNF-α and members of the IL-1 family) and angiogenic factors such as vascular endothelial growth factor A (VEGFA), are among the most extensively studied candidate biomarkers in translational research on cellular senescence and biological ageing (Coppé et al. 2010). In parallel, additional circulating factors frequently investigated in this context, including matrix metalloproteinases (MMPs), reflect broader inflammatory, tissue remodelling and stress-response pathways and exhibit limited specificity for senescent cell states. Other modalities, including circulating EVs, cell-free nucleic acids and molecular imaging approaches targeting SA-β-gal activity or senescence-associated cell-surface antigens, largely remain in preclinical or early-stage clinical development. Accordingly, contemporary biomarker development increasingly prioritises multimodal frameworks integrating molecular, cellular and functional indices. Rigorous analytical validation and prospective clinical qualification of such frameworks are essential prerequisites for robust evaluation of senotherapeutic interventions.

The absence of a universal biomarker underscores the need for multiparametric approaches. Current consensus frameworks recommend combining multiple markers from distinct categories alongside functional assays to improve confidence in senescence identification (Adewoye et al. 2020). Development of reliable and minimally invasive biomarkers remains a major unmet requirement for the clinical translation of senotherapeutics.

## Why senescent cells matter in ageing and disease

With advancing age, senescent cells accumulate across multiple tissues, including skin, bone, skeletal muscle, vasculature, liver, kidney, lung and brain, although the magnitude, cellular composition and kinetics of accumulation vary across tissues and cell types (Birch et al. 2015). Their persistence reflects both increased cellular stress burden and declining immune surveillance, particularly involving NK cells, macrophages and other components of the ageing immune system. Even a relatively small senescent cell fraction can exert disproportionate tissue effects because senescent cells may function as network hubs that propagate dysfunction across tissues (Nelson et al. 2012; da Silva et al. 2019). Through SASP signalling, ECM remodelling and extracellular vesicle (EV) release, local cellular damage can be amplified into broader inflammatory and regenerative dysfunction.

**Fig. 2:**
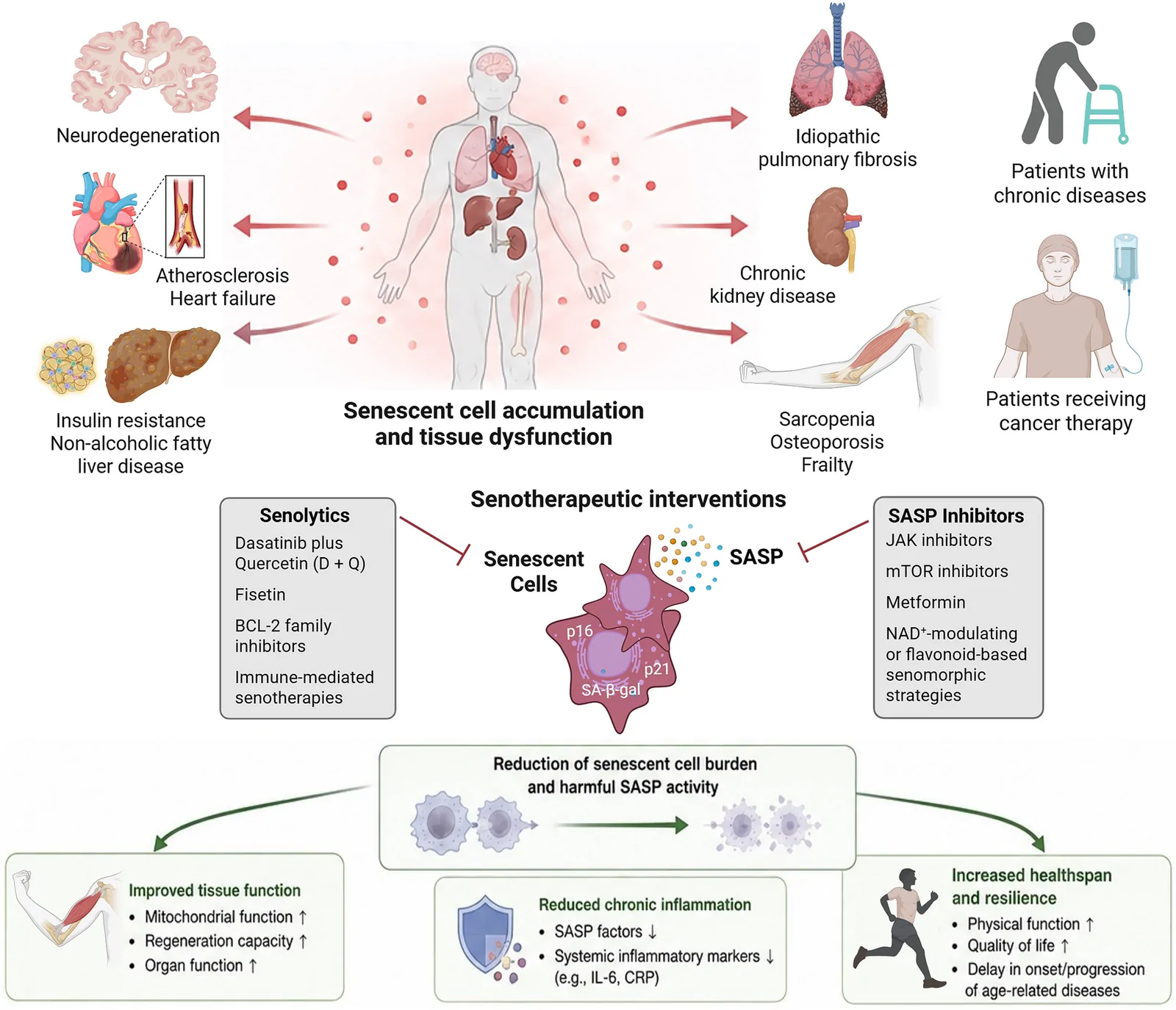
Cellular senescence, inflammageing and emerging senotherapeutic strategies in ageing and age-related disease. Cellular senescence is characterised by stable cell-cycle arrest and acquisition of a SASP. Age-related accumulation of senescent cells and persistent SASP signalling contribute to chronic inflammation, impaired tissue homeostasis and reduced regenerative capacity, and are associated with multiple age-related disorders, including cardiovascular, metabolic, pulmonary, musculoskeletal, neurodegenerative and neoplastic diseases. Senotherapeutic approaches, including senolytics and SASP inhibitors, are being investigated as mechanistically informed strategies to selectively reduce senescent cell burden and attenuate SASP signalling. These interventions may improve tissue function and resilience and potentially delay the onset or progression of selected age-related diseases; however, their long-term efficacy, safety and clinical applicability remain to be established. *BCL-2* B-cell lymphoma 2, *CRP* C-reactive protein, *JAK* Janus kinase, *mTOR* mechanistic target of rapamycin, *SASP* senescence-associated secretory phenotype

The contribution of cellular senescence to age-related disease is increasingly supported by experimental and clinical evidence (Fig. 2, Table 1). In atherosclerosis and vascular ageing, senescent endothelial and vascular smooth muscle cells contribute to endothelial dysfunction, plaque instability and arterial stiffening (Childs et al. 2016). In neurodegeneration, senescence-like phenotypes in astrocytes, microglia, oligodendrocyte lineage cells and potentially certain post-mitotic cell populations may amplify neuroinflammation, impair proteostasis and disrupt synaptic homeostasis (Bhat et al. 2012). In chronic kidney disease and idiopathic pulmonary fibrosis, cell senescence promotes maladaptive repair, ECM remodelling and disease progression. In osteoarthritis, senescent chondrocytes and stromal cells reinforce matrix degradation and inflammation associated with joint dysfunction (Peilin et al. 2019). In metabolic disease, cell senescence contributes to insulin resistance and chronic inflammation. In cancer, cellular senescence suppresses malignant transformation by enforcing stable growth arrest in damaged cells. However, therapy-induced or persistent senescence may promote immune evasion, tumour recurrence, fibrosis and treatment-related frailty when senescent cells are not efficiently cleared.

**Table 1:** Disease-relevant senescent cell populations, representative SASP mediators and emerging senotherapeutic strategies in major age-related disorders

Disease	Predominant senescent cell populations	Representative SASP factors and associated mediators	Senotherapeutic candidates	References
Age-related macular degeneration (AMD)	Retinal pigment epithelial cells, choroidal endothelial cells, Müller glia, senescent-like retinal microglia	VEGF-A, IL-8, TNF-α, CCL2, MMP-2/9, ICAM-1, complement activation (C3a/C5a)	D + Q, metformin procyanidin C1 (PCC1), nanoparticles	Chae et al. 2021; Lee et al. 2021; Nikolaidou et al. 2025
Alzheimer’s disease (AD)	Astrocytes, microglia, oligodendrocyte progenitor cells (OPCs), cerebrovascular endothelial cells, pericytes	C1q/C3, IL-1α, HMGB1, S100β, MMP-9, CCL2, YKL-40 (CHI3L1)	D + Q, fisetin, rapamycin/rapalogs, metformin, NAD^+^ precursors	Bussian et al. 2018; Zhang et al. 2019; Kuźniar et al. 2025
Parkinson’s disease (PD)	Dopaminergic neurons (senescence-like phenotype), astrocytes, microglia	Extracellular α-synuclein, IL-1β, CCL2, MMP-3, galectin-3, complement activation (C1q/C3)	D + Q, fisetin, rapamycin/rapalogs, NAD^+^ precursors	Chinta et al. 2013; Zaman et al. 2021; Wang et al. 2024a, b
Atherosclerosis	Vascular endothelial cells, vascular smooth muscle cells, macrophages/foam cells	VCAM-1, ICAM-1, E-selectin, IL-1β, CCL2, MMP-12, osteopontin, PAI-1	Statins, metformin, canakinumab	Longo et al. 2015; Ridker et al. 2017; Garrido et al. 2022
Heart failure (HF)	Cardiac fibroblasts, endothelial cells, vascular smooth muscle cells, cardiomyocytes (senescence-like phenotype)	GDF-15, galectin-3, sST2, TGF-β, CTGF, periostin, activin A, PAI-1, osteopontin	Rapamycin/rapalogs, metformin, D + Q, fisetin	Liberale et al. 2022; Puspitasari et al. 2022
Chronic kidney disease (CKD)	Tubular epithelial cells, podocytes, peritubular capillary endothelial cells, interstitial fibroblasts, pericytes	PAI-1, TGF-β, CTGF (CCN2), CCL2, galectin-3, activin A, osteopontin, KIM-1, NGAL	D + Q, fisetin, rapamycin/rapalogs	Wang et al. 2017; Morevati et al. 2026
Idiopathic pulmonary fibrosis (IPF)	Alveolar type II epithelial cells, fibroblasts, myofibroblasts, pulmonary endothelial cells, bronchiolar epithelial cells	TGF-β, CTGF (CCN2), PAI-1, activin A, MMP-7, periostin	D + Q, αvβ6 integrin-TGF-β activation blockade, FOXO4-DRI	Schafer et al. 2017; Merkt et al. 2020
Type 2 diabetes mellitus (T2DM)	Adipose progenitor/stromal cells, adipocytes, pancreatic β-cells, vascular endothelial cells, vascular smooth muscle cells	PAI-1, IL-1β, TNF-α, resistin, osteopontin, CXCL5	Metformin, D + Q, fisetin	Palmer et al. 2015; Palmer and Kirkland 2016; Burton and Faragher 2018

Experimental evidence from genetic and pharmacological studies supports a causal contribution of senescent cells to ageing and age-related pathology. In mice, genetic or pharmacological clearance of senescent cells improves tissue function and extends healthspan, whereas transplantation of relatively small numbers of senescent cells into young recipients induces physical dysfunction and accelerates features of ageing. Importantly, senescent-cell clearance initiated during later life can still improve tissue function, indicating that senescence is not merely a marker of accumulated damage but an active contributor to ongoing pathology. Collectively, these findings have transformed senescence from a descriptive feature of ageing into a mechanistically actionable therapeutic target and support a causal contribution of senescent cells to diverse age-related disorders.

Cardiovascular disease represents one of the most extensively investigated contexts in senescence research (Hu et al. 2022; Xu et al. 2025). Senescent endothelial and vascular smooth muscle cells accumulate within atherosclerotic lesions, where SASP factors contribute to plaque progression, instability and vascular dysfunction (Childs et al. 2016). SASP-derived MMPs degrade the fibrous cap, whereas inflammatory mediators, including IL-6, IL-8 and MCP-1 recruit immune cells and amplify local inflammation. Senescent adipocytes within perivascular adipose tissue may further impair vascular homeostasis through altered paracrine signalling and inflammatory mediator release. Following myocardial infarction, senescent cardiomyocytes and cardiac fibroblasts contribute to adverse ventricular remodelling, interstitial fibrosis and progressive cardiac dysfunction. Genetic or pharmacological clearance of senescent cells attenuates atherosclerotic burden and improves post-infarction cardiac function in aged mice (Zhu et al. 2013; Walaszczyk et al. 2019).

Metabolic dysfunction is closely linked to the accumulation of senescent cells in metabolically active tissues, where senescence-associated signalling contributes to chronic systemic inflammation and disruption of homeostasis. Senescent preadipocytes and adipocytes increase in visceral adipose tissue during ageing as well as obesity and secrete SASP factors, including IL-6, TNF-α, MCP-1 and PAI-1, which impair insulin signalling, promote chronic inflammation and facilitate immune-cell recruitment (Tchkonia et al. 2010; Ogrodnik et al. 2019). Senescent hepatocytes have been implicated in non-alcoholic fatty liver disease (NAFLD) and progression towards steatohepatitis, fibrosis and cirrhosis (Ogrodnik et al. 2017). In pancreatic islets, senescent β-cells exhibit impaired glucose-stimulated insulin secretion and may negatively affect neighbouring β-cell function through paracrine signalling (Aguayo-Mazzucato et al. 2019).

Neurodegenerative diseases are increasingly recognised as involving senescence-associated mechanisms (Bhat et al. 2012; Martínez-Cué and Rueda 2020). Senescence-like phenotypes in astrocytes, microglia and oligodendrocyte precursor cells increase with age and in disorders such as Alzheimer’s and Parkinson’s disease (Bussian et al. 2018; Chinta et al. 2018; Zhang et al. 2019; Martínez-Cué and Rueda 2020). Senescent astrocytes lose homeodynamic functions, including metabolic support, glutamate regulation and blood–brain barrier maintenance, while acquiring pro-inflammatory phenotypes. Senescent microglia display impaired phagocytic function and sustained inflammatory signalling that may contribute to neuroinflammation and synaptic dysfunction. In mouse models, genetic clearance of senescent cells reduces neurodegenerative phenotypes and preserves cognitive performance, providing evidence for a contributory role of cellular senescence in neurodegeneration (Ogrodnik et al. 2021).

Musculoskeletal disorders, including sarcopenia, osteoarthritis and osteoporosis, are also associated with senescent cell accumulation. Senescent satellite cells exhibit impaired regenerative capacity and contribute to age-related muscle dysfunction. In osteoarthritis, senescent chondrocytes secrete inflammatory mediators and MMPs that promote matrix degradation and propagate local senescence signalling. Senescent osteocytes and osteoblasts can impair bone homeostasis by promoting osteoclast activity and suppressing bone formation. Senolytic interventions improve muscle function and preserve bone mass in preclinical studies.

Cancer exhibits a complex and context-dependent relationship with cellular senescence (Yang et al. 2021). Oncogene-induced senescence and DNA damage-induced senescence act as intrinsic tumour-suppressive mechanisms that constrain progression of premalignant lesions (Bartkova et al. 2006). However, persistent senescent cells can also promote tumorigenesis through SASP-mediated effects. GFs, inflammatory mediators and matrix-remodelling enzymes may generate a pro-tumorigenic microenvironment that supports proliferation, angiogenesis, epithelial–mesenchymal transition and metastatic progression. Therapy-induced senescence, a frequent consequence of chemotherapy and radiotherapy, may generate a persistent senescent cell burden that contributes to treatment-related toxicity and tumour recurrence. Consequently, combination strategies incorporating senotherapeutics alongside conventional cancer therapies are under active investigation.

## Targeting cellular senescence: emerging strategies and translational opportunities

Translation of insights from senescence biology into therapeutic intervention has accelerated substantially and now encompasses genetic, immunological, pharmacological and cell-based approaches. These strategies aim to eliminate senescent cells in a controlled manner (senolytics), attenuate their deleterious secretory activity (senomorphics) and enhance endogenous immune-mediated mechanisms that limit senescent-cell persistence.

Genetic clearance models, although not directly translatable to humans, have provided essential mechanistic insight and proof-of-concept evidence. The INK-ATTAC transgenic mouse model expresses a drug-inducible caspase-8 fusion protein under control of the p16INK4a promoter, enabling selective elimination of p16-expressing cells following administration of a synthetic dimerising compound (AP20187). Intermittent clearance of senescent cells delays multiple age-associated pathologies and improves healthspan in mice (Baker et al. 2011; 2016; Chang et al. 2016).

Immunological approaches seek either to engineer immune cells or enhance physiological senescent-cell clearance mechanisms. Chimeric antigen receptor (CAR) T cells directed against senescence-associated surface antigens represent one such strategy. Proof-of-concept studies demonstrated that CAR T cells targeting urokinase plasminogen activator receptor (uPAR), which is upregulated in several senescent cell populations, can eliminate senescent cells, improve metabolic homeostasis and reduce hepatic pathology in mice (Amor et al. 2020). Additional candidate targets, including B7-H3 and selected NOTCH-related pathways, are under investigation. Potential advantages include cellular specificity, persistence and sustained immune surveillance. However, clinical translation is constrained by the absence of truly senescence-specific antigens and by inter-population heterogeneity of senescence-associated markers, which increase the risk of on-target off-tissue cytotoxicity and may reduce therapeutic efficacy. Further constraints include CAR-T manufacturing complexity, cytokine-mediated toxicities and potential immune-related adverse events.

Enhancing endogenous immune-mediated clearance offers an alternative strategy. Physiological removal of senescent cells is mediated largely by natural killer (NK) cells, which recognise stress-induced ligands including MICA, MICB and ULBP family proteins and induce apoptosis through death-receptor pathways such as TRAIL signalling. Macrophages also contribute through recognition of pro-phagocytic signals such as calreticulin exposure and reduced expression of anti-phagocytic signals including CD47.

Ageing is associated with impaired NK-cell cytotoxicity and altered macrophage function, potentially reducing senescent-cell clearance efficiency. Consequently, interventions designed to augment innate immune surveillance, including IL-15-based approaches that expand NK-cell populations, anti-CD47 therapies that promote phagocytosis and emerging senescence-directed vaccination strategies, are under active investigation. Preclinical studies suggest that these approaches may reduce senescent-cell burden and improve functional outcomes in aged mice.

Drug repurposing has further accelerated senotherapeutic development. Metformin, a widely prescribed anti-diabetic drug and AMPK activator, can attenuate some aspects of the SASP partly through modulation of AMPK-mTOR-NF-κB signalling and has been associated with improved healthspan in experimental and preclinical studies (Anisimov et al. 2008; Algire et al. 2012; Hu et al. 2020). Rapamycin and rapalogs may suppress mTOR signalling and attenuate SASP activity (Harrison et al. 2009). Ruxolitinib and other Janus kinase (JAK) inhibitors can reduce inflammatory signalling downstream of SASP-associated cytokines without directly eliminating senescent cells (Xu et al. 2015). These repurposed agents benefit from established safety profiles, well-characterised pharmacokinetics and existing regulatory frameworks that may facilitate clinical translation. Collectively, these approaches considerably broaden the therapeutic landscape of senescence-targeting interventions and may ultimately support complementary or combinatorial strategies addressing multiple dimensions of senescence biology.

## Senolytics and SASP modulation: from experimental promise to clinical translation

Pharmacological senotherapeutics are generally classified as senolytics, which selectively eliminate senescent cells, and senomorphics (SASP inhibitors, senostatics), which attenuate SASP activity without inducing cell death (Fig. 2). Both classes have progressed into early clinical evaluation but continue to face important translational challenges. Senolytic agents exploit the apoptosis-resistant phenotype of senescent cells, which frequently depends upon activation of pro-survival pathways including members of the BCL-2 protein family (Zhu et al. 2016).

Pharmacological disruption of these pathways can preferentially trigger apoptosis in senescent cells. Dasatinib plus quercetin (D + Q) remains the most extensively investigated senolytic combination (Hickson et al. 2019). Dasatinib preferentially targets senescent preadipocytes and endothelial cells through inhibition of multiple tyrosine kinase pathways, whereas quercetin exhibits broader activity involving PI3K/AKT and anti-apoptotic signalling pathways. Together, these agents display complementary senolytic activity across distinct senescent-cell populations. In aged mice, intermittent D + Q treatment reduces senescent-cell burden, lowers circulating SASP factors and improves measures of physical and vascular function. In an initial pilot study involving patients with diabetic kidney disease, D + Q treatment reduced adipose tissue expression of p16^INK4a^ and p21^CIP1/WAF1^ and lowered circulating SASP factors. Several studies on idiopathic pulmonary fibrosis have reported improvements in physical function and reductions in selected SASP markers. Ongoing clinical studies are evaluating D + Q in conditions including chronic kidney disease, Alzheimer’s disease, frailty and osteoarthritis (Jeon et al. 2017; Hickson et al. 2019; Ogrodnik et al. 2021).

Navitoclax (ABT-263) and related compounds were originally developed as anti-cancer agents targeting BCL-2 family proteins (Zhu et al. 2016; González-Gualda et al. 2020). These agents demonstrate potent senolytic activity in haematopoietic stem cells, endothelial cells and fibroblasts and improve functional outcomes in preclinical ageing models. However, thrombocytopenia resulting from platelet dependence on BCL-xL remains a major limitation. Consequently, efforts are focused on developing more selective delivery systems and dosing strategies to reduce toxicity.

Fisetin, a naturally occurring flavonoid found in various fruits (e.g. strawberries, apples) and vegetables, has also demonstrated senolytic activity in animal models. In mice, for example, fisetin treatment reduces inflammatory burden and improves healthspan measures (Yousefzadeh et al. 2018). Early clinical studies suggest acceptable safety profiles, although evidence for clinical efficacy remains limited. Additional strategies aim to improve targeting specificity. These include HSP90 inhibition and prodrug systems activated by senescence-associated β-galactosidase activity, which exploit elevated lysosomal activity in senescent cells to preferentially release cytotoxic agents within target cells.

Senomorphics attenuate SASP production or downstream signalling while preserving cell viability. This approach may be advantageous when transient senescence fulfils physiological functions or when extensive cell elimination could disrupt tissue homeostasis. Rapamycin and rapalogs suppress mTORC1 signalling, a central regulator of SASP production (Herranz et al. 2015). Experimental studies demonstrate reductions in secretion of inflammatory cytokines and matrix-remodelling factors, alongside improvements in immune function and lifespan extension in model organisms. However, chronic mTOR inhibition may produce adverse metabolic effects requiring careful optimisation of treatment regimens.

JAK inhibitors, including ruxolitinib and baricitinib, attenuate signalling downstream of SASP-associated cytokines and reduce inflammatory activity without directly removing senescent cells (Xu et al. 2015). Their existing clinical use provides opportunities for therapeutic repurposing. Targeted anti-inflammatory approaches are also being explored. Interventions targeting IL-1β, IL-6, TNF-α and NLRP3 signalling may attenuate selected components of senescence-associated inflammation (Furman et al. 2019). The CANTOS trial provided proof-of-concept evidence that selective inhibition of IL-1β signalling can reduce recurrent cardiovascular events, with subsequent analyses linking clinical benefit to on-treatment reductions in inflammatory biomarkers (Ridker et al. 2017; 2018).

Despite considerable progress, substantial challenges remain. Senolytic effects may be transient because senescent cells continue to arise throughout life, making treatment schedule optimisation an unresolved issue. The lack of validated non-invasive biomarkers limits patient stratification, monitoring and endpoint assessment. Moreover, marked heterogeneity across tissues, disease states and individuals suggests that future interventions may require biomarker-guided, tissue-specific and personalised approaches.

Long-term safety also remains uncertain. Excessive suppression of senescence could impair physiological processes including tissue repair and tumour suppression, whereas broad inhibition of SASP activity may alter immune surveillance and tissue remodelling. In addition, long-term or repeated senotherapeutic exposure may carry context-dependent risks that differ across tissues, particularly in regenerative compartments where senescence contributes to wound resolution and developmental-like repair programmes. Combination approaches may therefore offer greater therapeutic potential than single-agent interventions. Integration of senotherapeutics with interventions targeting additional hallmarks and mechanisms of ageing, including mitochondrial function, immune regulation and proteostasis, may ultimately provide more effective and context-dependent strategies (Chmielewski 2026). Development of reliable biomarkers, including circulating SASP signatures, extracellular vesicle profiling, senescence-associated methylation patterns and molecular imaging approaches, remains a major priority for clinical translation.

## Extracellular vesicles (EV) as markers, mediators and therapeutic targets

EVs, including exosomes and microvesicles, are increasingly recognised as key mediators of intercellular communication that contribute to the propagation of senescence-associated and inflammatory signalling beyond the local tissue environment (Takasugi 2018; Wallis et al. 2020; Oh et al. 2022). Senescent cells release EVs with altered molecular cargo comprising miRNAs, proteins, lipids, metabolites and nucleic acids that can affect neighbouring and distant cells (Estévez-Souto et al. 2023).

EV-mediated communication may help explain how local cellular damage generates broader tissue dysfunction. Senescent cell-derived EVs carry inflammatory and pro-fibrotic mediators capable of inducing paracrine senescence, amplifying inflammatory signalling and modifying ECM remodelling in recipient cells (Hou et al. 2024). Several senescence-associated miRNAs, including miR-21, miR-34a and miR-146a, have been implicated in these processes, although EV cargo composition varies substantially according to cell type and senescence-inducing stimulus (Estévez-Souto et al. 2023).

The properties of EVs have generated interest in their therapeutic potential. One strategy aims to suppress deleterious signalling by reducing secretion or uptake of senescence-associated EVs. Inhibition of EV biogenesis or release, including approaches targeting neutral sphingomyelinase activity, has attenuated paracrine senescence and inflammatory phenotypes in experimental systems (Estévez-Souto et al. 2023). However, the broad physiological functions of EVs suggest that indiscriminate inhibition can produce unintended consequences.

An alternative strategy seeks to exploit EVs as therapeutic delivery systems. EVs derived from young, healthy or stem-cell populations frequently display anti-inflammatory and regenerative properties and may transport bioactive cargo capable of modulating tissue repair. Mesenchymal stem cell-derived EVs have shown beneficial effects in preclinical studies, including reduced apoptosis, attenuation of inflammatory signalling and improvement of tissue function following injury (Grigorian Shamagian et al. 2023). Engineered EVs also offer potential as targeted delivery platforms for nucleic acids, proteins and small molecules, exploiting intrinsic tissue tropism and biocompatibility (Romero-García et al. 2023).

At present, EVs show greatest translational promise as liquid biopsy biomarkers, although their potential as therapeutic delivery vehicles and interventional targets remains under active investigation (Chen et al. 2024; Yang et al. 2024; Chmielewski et al. 2025). Because EVs can be isolated from blood, urine and cerebrospinal fluid (CSF), they represent attractive candidates for minimally invasive assessment of tissue state, biological ageing and therapeutic response. Distinct EV-associated molecular signatures, including specific miRNAs and long non-coding RNAs, have shown diagnostic and prognostic potential across cardiovascular, neurodegenerative and metabolic diseases.

Despite considerable promise, several obstacles continue to impede clinical translation. Isolation and purification remain incompletely standardised, cargo characterisation is inconsistent across studies and robust potency assays are still lacking. Dose optimisation and biodistribution also remain unresolved. Accordingly, although EVs might become a useful addition to the senotherapeutic arsenal, their most immediate translational value is likely to lie in biomarker-guided stratification and targeted delivery within broader therapeutic strategies.

## Ageing as unresolved tissue injury: towards resolution-based interventions

**Fig. 3:**
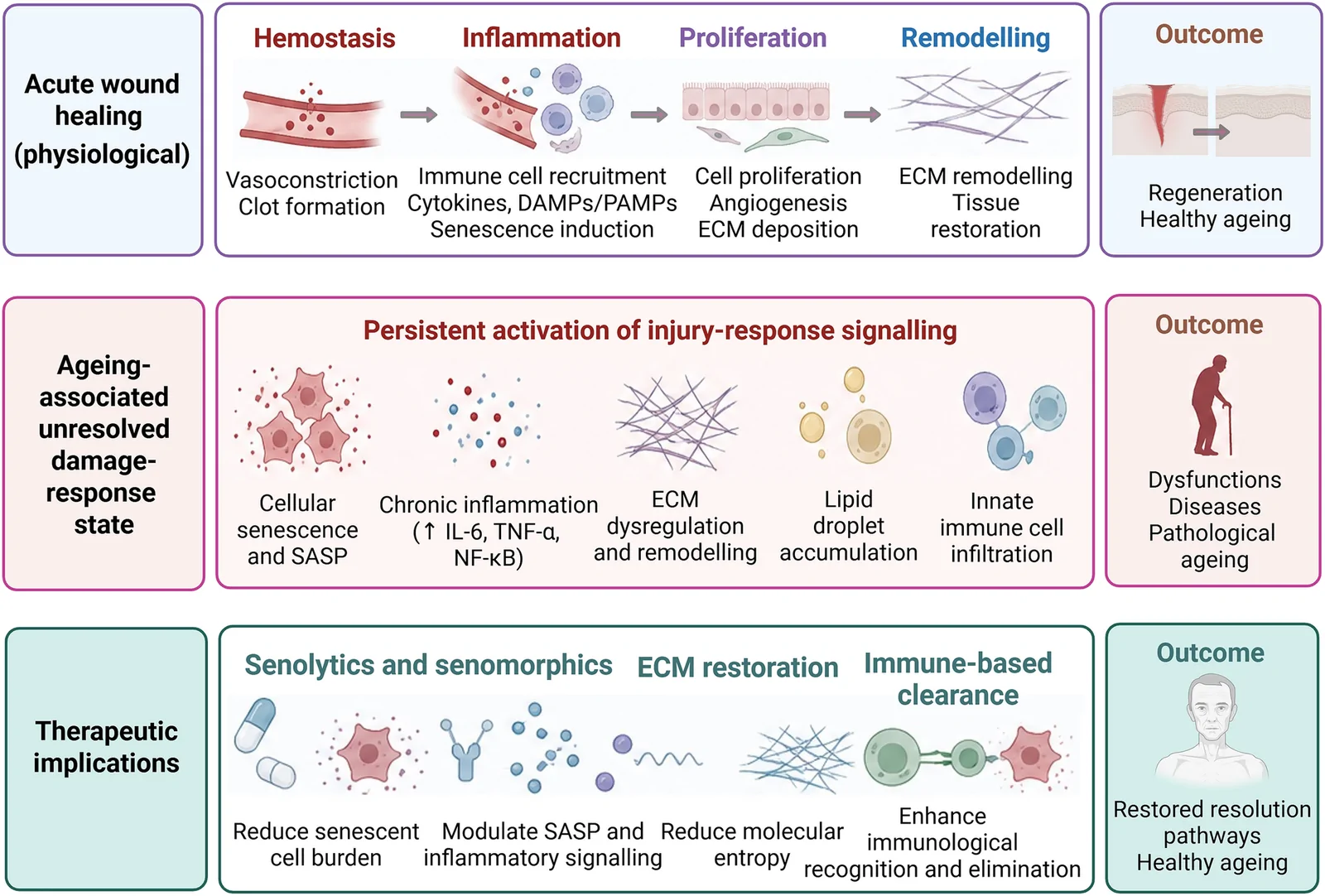
Ageing as persistent unresolved injury-response signalling. Ogrodnik (2025) recently proposed that biological ageing may reflect chronic persistence of tissue responses normally associated with the inflammatory phase of wound healing. Senescent cells reinforce this unresolved repair state through persistent SASP-mediated inflammatory and matrix-remodelling signalling. This framework suggests that pathological ageing may emerge not only from cumulative molecular damage, but also from impaired termination of evolutionarily conserved repair-associated signalling pathways. Consequently, effective gerotherapeutics may require restoration of resolution biology and tissue homeostasis in addition to suppression of senescent-cell burden and chronic systemic inflammation. *DAMPs* damage-associated molecular patterns; *ECM* extracellular matrix, *NF-κB* nuclear factor κB, *PAMPs* pathogen-associated molecular patterns, *SASP* senescence-associated secretory phenotype

The recent proposal that biological ageing reflects the maladaptive persistence of tissue damage-response states (Ogrodnik 2025) offers a compelling system-level framework for geroscience (Fig. 3). In this view, many aged tissues retain features of the inflammatory phase of wound healing but fail to transition efficiently to resolution, regeneration and structural remodelling. Chronic inflammation, ECM dysregulation, immune infiltration, lipid-droplet accumulation and cellular senescence may therefore be interpreted not as independent hallmarks but as coordinated manifestations of persistently engaged tissue damage responses.

Senescent cells occupy a central position in this framework. As both sensors and amplifiers of unresolved damage, they sustain the SASP, maintaining inflammatory and matrix-remodelling signals that are normally transient during acute repair. This perspective addresses a long-recognised paradox in ageing biology: interventions that attenuate growth-promoting and repair-associated signalling frequently extend lifespan in animal models, whereas persistent wound-healing-like activity accelerates late-life pathology (Ogrodnik 2025). From an evolutionary perspective, this interpretation is consistent with antagonistic pleiotropy, whereby mechanisms that enhance early-life survival and tissue repair incur late-life costs when chronically engaged (Williams 1957; Chmielewski 2017; Long and Zhang 2023). Ageing may therefore be interpreted not merely as progressive decline in repair and maintenance capacity, leading to accumulation of molecular and cellular damage and tissue dysfunction, but as failure to resolve tissue damage-response states that are normally self-limiting following acute injury (Ogrodnik 2025).

Therapeutically, this perspective suggests that senolytics, senomorphics and immune-mediated senescent-cell clearance strategies, although valuable, may prove insufficient in isolation. Durable rejuvenation may instead require restoration of endogenous resolution systems, including immune recalibration, extracellular matrix homeostasis, stem-cell niche integrity and regeneration-supportive signalling networks.

Ageing as dysregulated tissue repair: open questions and future directions.

Current frameworks provide mechanistic insight into many molecular and cellular hallmarks of ageing but do not yet fully explain how these processes collectively give rise to the progressive deterioration of tissue architecture, regenerative capacity and organ function across tissues and over time (López-Otín et al. 2013, 2023). Emerging frameworks propose that these higher-order phenotypes arise from dysregulated dynamics of tissue repair responses, including persistent engagement, repeated initiation and incomplete resolution following activation (Ogrodnik 2025). Following acute injury, tissues initiate evolutionarily conserved, temporally ordered and spatially constrained repair responses encompassing inflammation, ECM remodelling, debris clearance, metabolic reprogramming and transient SASP signalling (Rodier and Campisi 2011; Muñoz-Espín et al. 2013; Muñoz-Espín and Serrano 2014). This framework provides a basis for understanding why adult mammalian repair commonly proceeds through fibrosis and scarring, whereas robust regeneration are largely restricted to embryonic development, specific adult tissues and regenerative species such as axolotls (*Ambystoma mexicanum*).

**Fig. 4:**
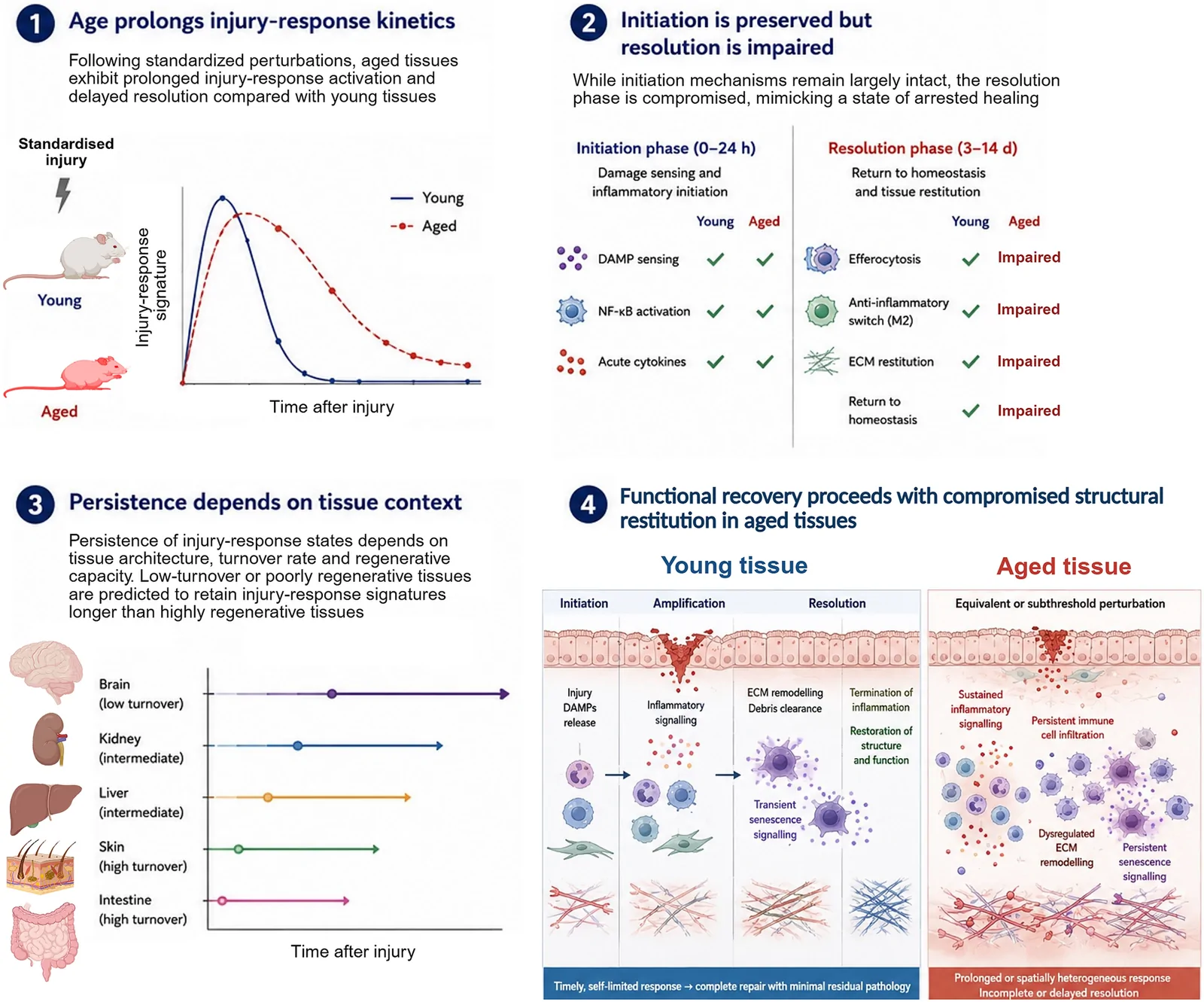
Testable predictions of Ogrodnik’s tissue repair framework for ageing

Within this framework, ageing is viewed as a dynamic process in which tissue repair responses are incompletely resolved, aberrantly sustained or re-initiated following persistent or cumulative tissue perturbation (Fig. 3). The overlap between acute injury responses and ageing phenotypes, including chronic inflammation, immune cell infiltration, ECM remodelling, metabolic reprogramming, lipid droplet accumulation and SASP activity, suggests that many features of ageing reflect dysregulated resolution dynamics of conserved repair responses operating under altered temporal and spatial constraints. Importantly, this framework yields experimentally testable predictions spanning molecular, cellular, tissue, organ and organismal levels of biological organisation (Fig. 4).

However, several fundamental questions remain incompletely resolved at both mechanistic (proximate) and evolutionary (ultimate) levels. First, which molecular and tissue-derived signals govern the sustained engagement, inappropriate activation or impaired resolution of tissue repair responses during ageing? Second, to what extent does ageing reflect persistent activation of these responses, defective termination following their initiation, or repeated cycles of activation and resolution failure across the lifespan? Third, how do tissue-specific repair architectures and microenvironmental constraints shape organ-specific trajectories of ageing in a quantitatively predictive manner? Finally, how have evolutionary trade-offs between rapid wound closure, regenerative fidelity and tumour suppression constrained the diversification of repair strategies across tissues and species? Addressing these questions will require integrated multi-scale, comparative and perturbational approaches that link molecular lesions and cell-state dynamics to tissue architecture, repair outcomes and physiological function.

## Conclusions

Cellular senescence exemplifies how an evolutionarily conserved and context-dependent stress-response mechanism can become deleterious when persistent or dysregulated. Its inflammatory and matrix-remodelling activities link genomic instability, organelle dysfunction, impaired tissue repair and maladaptive remodelling to systemic age-related dysfunction, including vascular disease, neurodegeneration, fibrosis, metabolic dysfunction and cancer progression. The key question is no longer whether cellular senescence matters, but which senescent states matter, in which tissues, and at which stages of ageing and disease, and when they should be modulated, eliminated or preserved. In this framework, senotherapeutics are not a single class of ‘anti-ageing’ drugs, but a set of precision strategies aimed at restoring tissue function and resilience. Senolytics, senomorphics and immune-mediated clearance approaches each target distinct constraints within senescence biology. Their success will depend on rigorous state definition, validated biomarkers, improved targeting and adequately powered clinical trials prioritising meaningful functional outcomes. If these conditions are met, senescence biology could support a transition in geroprevention analogous to that achieved by molecular oncology in cancer, shifting from empirical intervention towards mechanism-based, context-specific precision medicine.

## Data Availability

No datasets were generated or analysed during the current study.
